# Updating thermal imaging dataset of hand gestures with unique labels^☆^

**DOI:** 10.1016/j.dib.2022.108037

**Published:** 2022-03-08

**Authors:** Sreenivasa Reddy Yeduri, Daniel Skomedal Breland, Om Jee Pandey, Linga Reddy Cenkeramaddi

**Affiliations:** a ACPS Group, Department of Information and Communication Technology, University of Agder, Norway; b Department of Electronics Engineering, IIT(BHU), Uttar Pradesh 221005, India

**Keywords:** Thermal imaging, Hand Gestures, Thermal Camera, Machine learning models, Sensor

## Abstract

An update to the previously published low resolution thermal imaging dataset is presented in this paper. The new dataset contains high resolution thermal images corresponding to various hand gestures captured using the FLIR Lepton 3.5 thermal camera and Purethermal 2 breakout board. The resolution of the camera is 160×120 with calibrated array of 19,200 pixels. The images captured by the thermal camera are light-independent. The dataset consists of 14,400 images with equal share from color and gray scale. The dataset consists of 10 different hand gestures. Each gesture has a total of 24 images from a single person with a total of 30 persons for the whole dataset. The dataset also contains the images captured under different orientations of the hand under different lighting conditions.

**Specifications Table** This section list the details of the hardware, procedure for collecting the data, and the format of the data.

**Table:** 

Subject	Human-Computer Interaction, Biomedical, Electrical and Electronic Engineering
Specific subject area	Thermal images of different hand gestures
Type of data	Image (.png)
How data were acquired	Thermal Camera (Flir Lepton 3.5 thermal camera)
	Camera Stand
	Purethermal 2 breakout board
	Raspberry Pi 4 Model B
	Same as in original data article
Data format	Raw (from acquisition)
Parameters for data collection	Images are collected from 30 people with 160×120 pixel camera with a radiometric calibrated array of 19200 pixels
Description of data collection	The camera setup is mounted on a tripod to capture the images. Further, hand gestures are captured while hand is mostly static position. We have placed both camera setup and hand on top of a table to capture effective images. The software program was designed to save images based on the number that is being pressed as the first input in the range 1 to 5. Then, the second input is given to define the total number of images to be captured.
Data source location	ACPS group, Department of Information and Communication Technology, University of Agder, Grimstad, Norway
Data accessibility	Repository Name:
	Plain_Background_Thermal_Imaging_Dataset
	https://zenodo.org/record/6247463#.YhZ2gujMJaQ
Related data article	Sreenivasa Reddy Yeduri, Daniel Skomedal Breland, Simen Birkeland Skriubakken, Om Jee Pandey, Linga Reddy Cenkeramaddi, Low Resolution Thermal Imaging Dataset of Sign Language Digits, Data in Brief, 2022, 107977, ISSN 2352-3409, https://doi.org/10.1016/j.dib.2022.107977
Related research article	D. S. Breland, A. Dayal, A. Jha, P. K. Yalavarthy, O. J. Pandey and L. R. Cenkeramaddi, ”Robust Hand Gestures Recognition Using a Deep CNN and Thermal Images,” in IEEE Sensors Journal, vol. 21, no. 23, pp. 26602-26614, 1 Dec.1, 2021, doi: 10.1109/JSEN.2021.3119977

## Value of the Data

• The existing dataset contains the images of 32×32 pixel thermal camera [1], [2]. However, the new dataset is created with 160×120 pixel thermal camera.
• Efficient machine learning models can be developed to process the data for hand gesture recognition.
• The academic or research communities working on thermal imaging data with efficient machine learning algorithms for hand gesture recognition or classification.
• The data is also helpful in developing and testing efficient algorithms to work on thermal imaging dataset.
• The data is collected with high thermal camera with no constraints on the environment and captured images are independent of back ground lighting conditions. This will be helpful for testing the algorithms with thermal imaging data.

## 1 Data Description

The previous dataset in [1], [2] is captured with a low resolution thermal camera of 32×32 pixels resolution. The thermal images in this dataset correspond to ten hand gestures representing 0 to 9 sign language digits. This dataset has been created from various people with different hand orientations.

On the other hand, the dataset present in this paper contains the images captured from the thermal camera with the resolution of 160×120 pixels. The thermal images contain ten different hand gestures captured from various people. We also captured images of both color and gray scale under varying environment conditions. Further, different hand orientations are also considered while creating the dataset.

### 1.1 Data file description

**Fig. 1:**
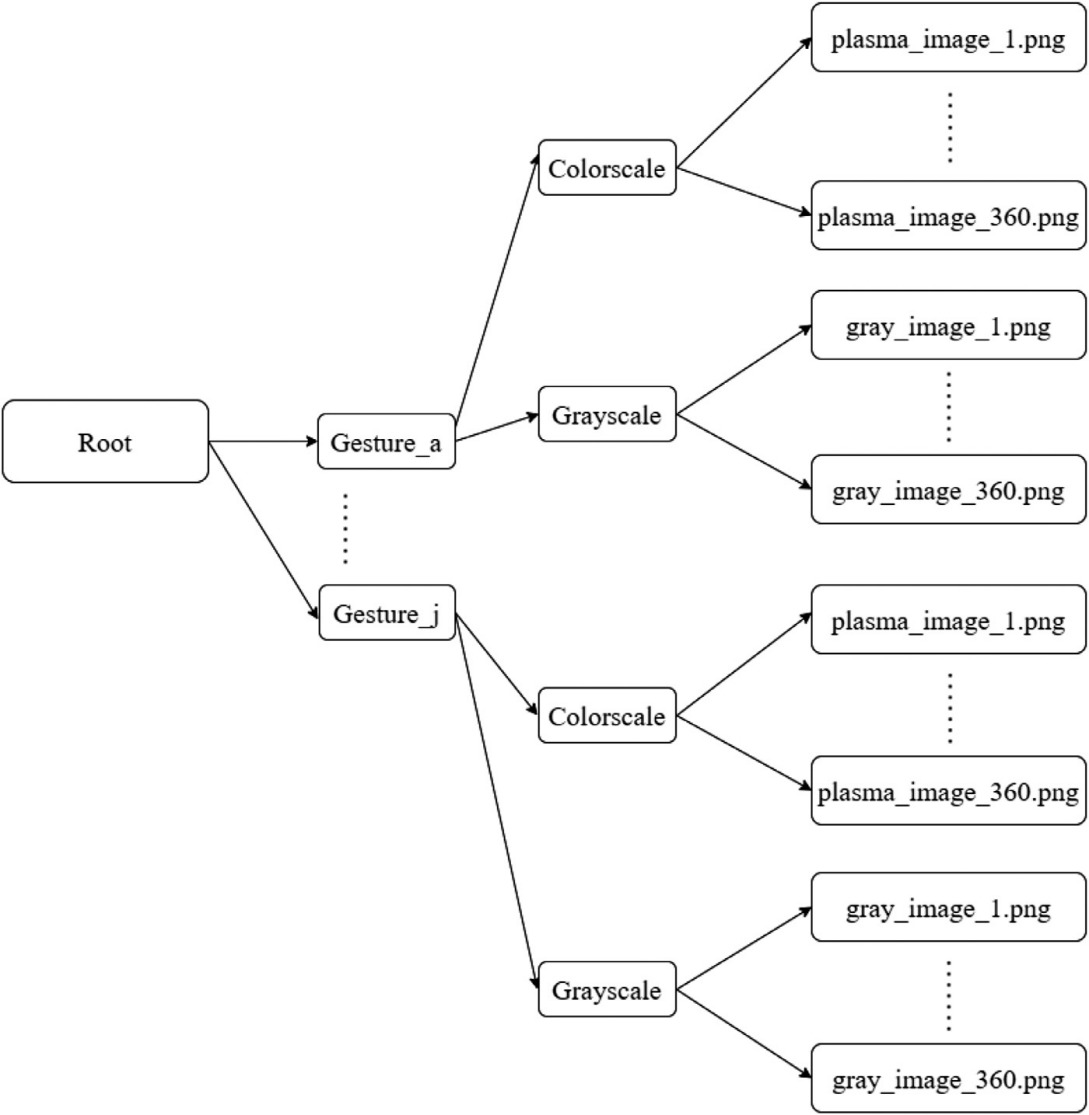
Data structure of the repository.

The data repository structure is shown in Fig. 1. The root folder consists of ten folders namely Gesture_a to Gesture_j corresponding to Gesture a to j, respectively. Further, each gesture folder contains two folders such as Colorscale and Grayscale. The Colorscale folder consists of 360 color images of.png format. Similarly, Grayscale folder consists of 360 gray images in.png format. There are a total of 30 people considered for the creation of dataset. The total size of the dataset is 154 MB [3].

**Fig. 2:**
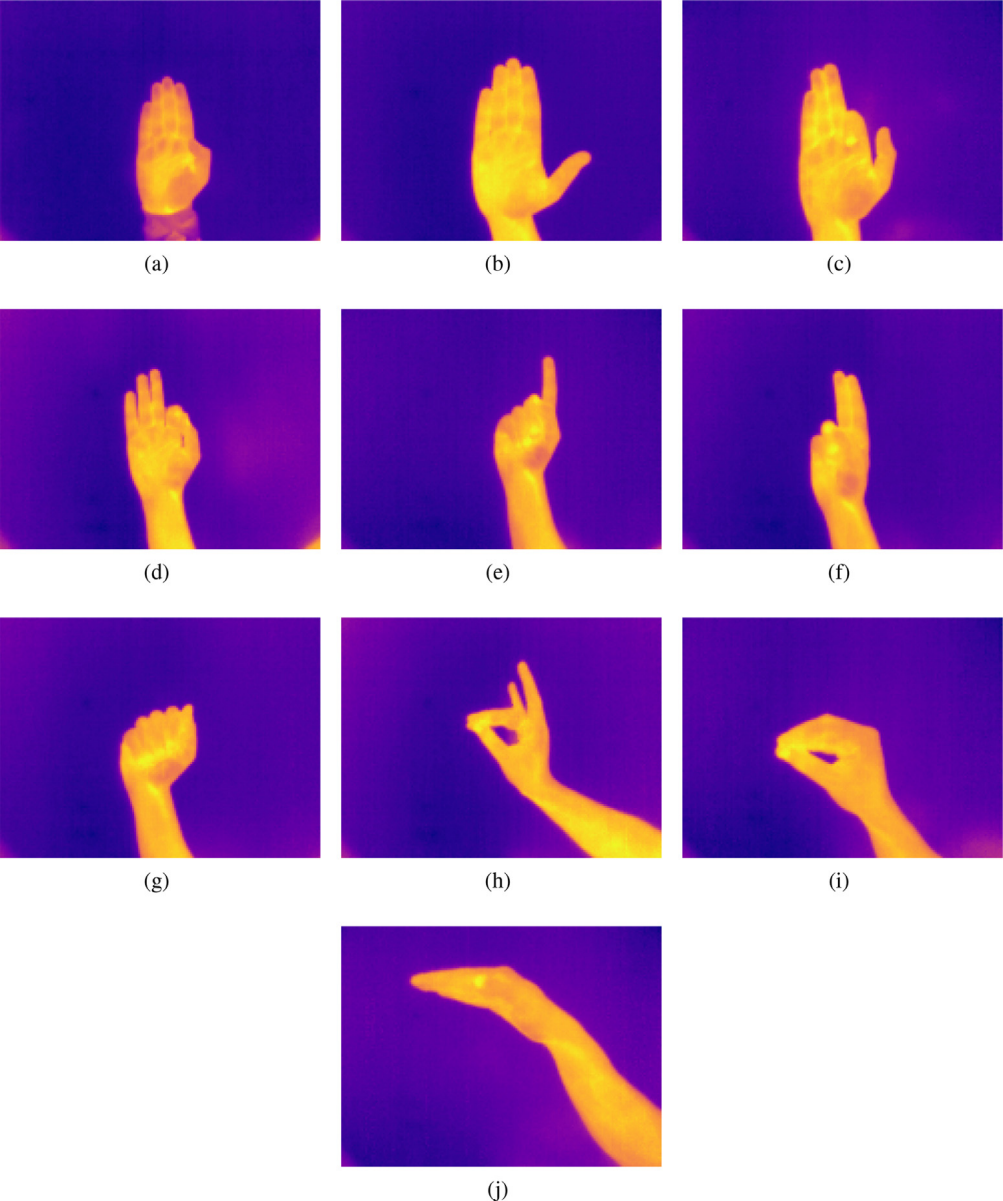
A colored fusion thermal images: (a) Image a; (b) Image b; (c) Image c; (d) Image d; (e) Image e; (f) Image f; (g) Image g; (h) Image h; (i) Image i; and, (j) Image j.

Fig. 2 shows the plasma thermal images corresponding to hand gestures from a to j. Fig. 2a is corresponding to image a and Fig. 2b corresponds to image b. Fig. 2c, 2d, 2e, 2f, 2g, 2h, 2i, and 2j are the color scale thermal images c, d, e, f, g, h, i, and j, respectively.

**Fig. 3:**
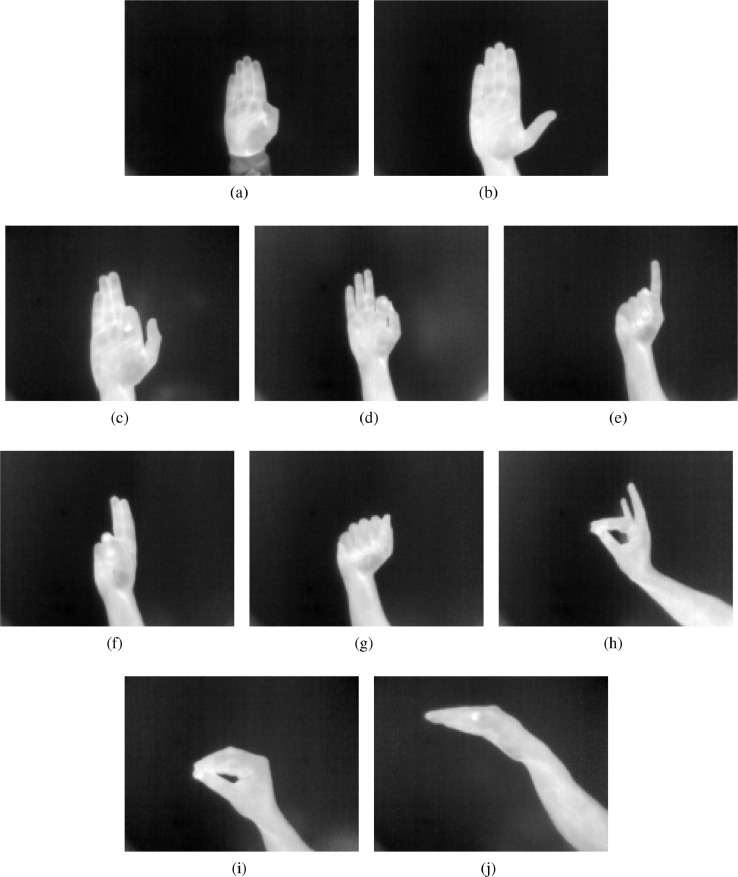
A gray fusion thermal images: (a) Image a; (b) Image b; (c) Image c; (d) Image d; (e) Image e; (f) Image f; (g) Image g; (h) Image h; (i) Image i; and, (j) Image j.

Fig. 3 shows the gray thermal images corresponding to hand gestures from a to j. Fig. 3a is corresponding to image a and Fig. 3b corresponds to image b. Fig. 3c, 3d, 3e, 3f, 3g, 3h, 3i, and 3j are the gray scale thermal images c, d, e, f, g, h, i, and j, respectively.

## 2 Experimental Design, Materials and Methods

**Fig. 4:**
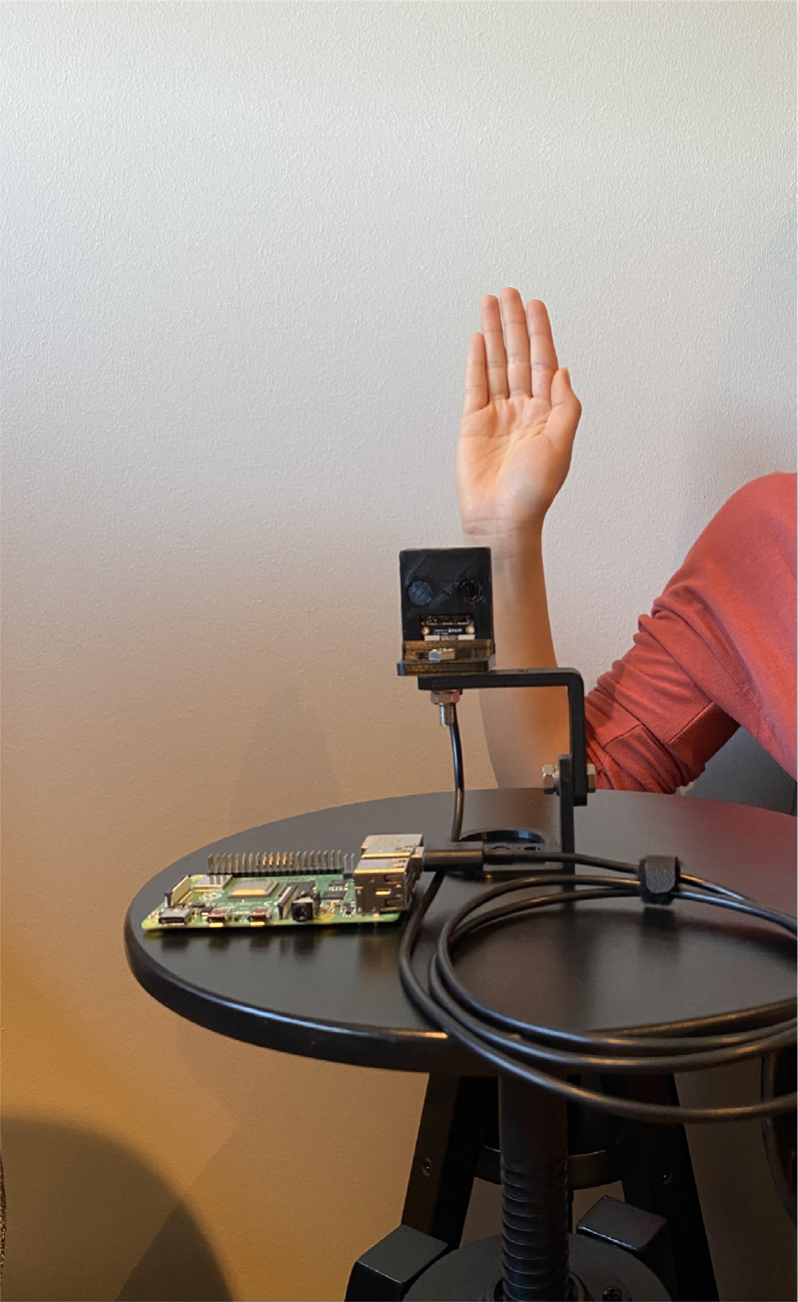
Thermal camera setup.

Fig. 4 shows the experimental setup of the thermal camera considered for the data collection. We used FLIR Leptop 3.5 thermal camera module to be fitted in embedded systems [4]. It has a horizontal field of view of 57${}^{\circ }$ which indicates that it captures more of image details than object details. The images can be captured by connecting the thermal camera to a computer with Windows OS and SDK [5]. A portable and simple setup is to connect the thermal camera to Raspberry Pi 4 Model B [6] which make use of the python script to capture the images. For the creation of the dataset, we connected the thermal camera to Raspberry Pi 4 model B.

The thermal camera do not have a port to directly connect to the Raspberry Pi. Thus, we fitted the camera on purethermal 2 breakout board [7], [8] which has an interface to connect to the Raspberry Pi through USB. Thereafter, we placed the breakout board on a fixed stand to stable the camera as well as to add height for a easy capturing of the hand gestures.

**Fig. 5:**
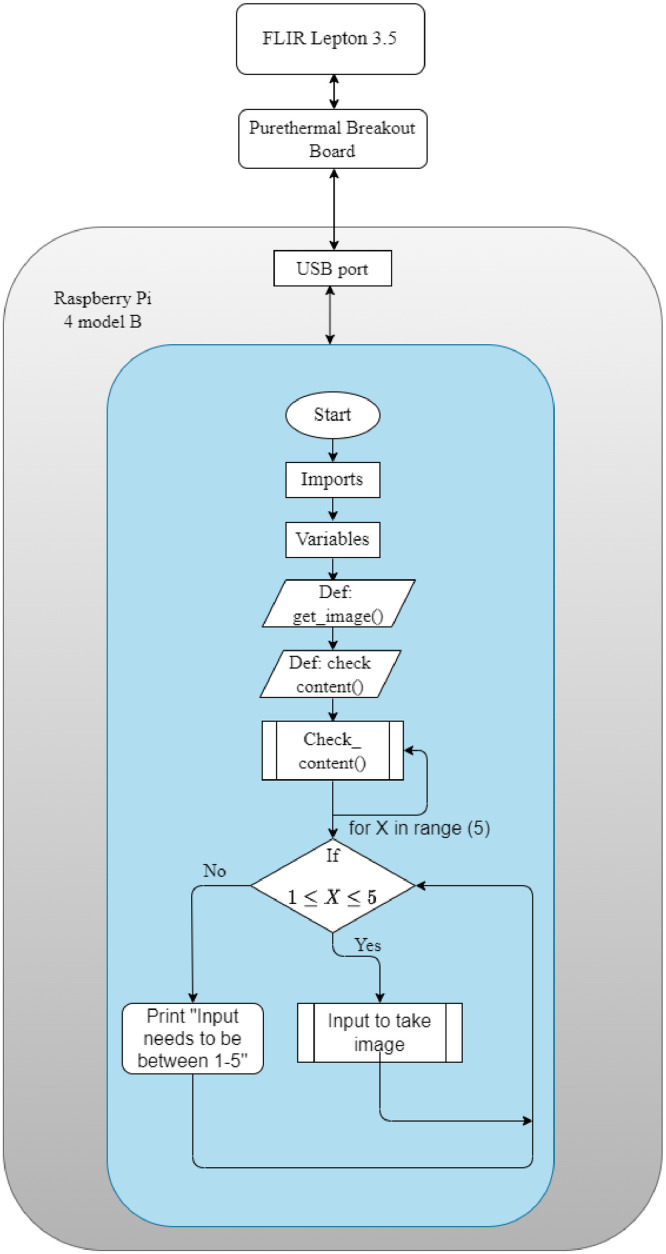
Thermal image capturing procedure.

Fig. 5 shows the steps in the python script to capture the images for the dataset. The program makes use of the Lepton library from flirpy in Python. This library enables the thermal camera for capturing the images and then the OpenCV and matplotlib libraries are used to save the images in the Raspberry Pi. The main loop takes the inputs from 1 to 5 to capture the images. When 2 is the input, it will take second input. The second input defines how many images the thermal camera should capture before asking for the first input again.

## Ethics Statement

The data consists solely of hand gestures and contains no personal information. It was a free-for-all campaign, and people gave the hand gestures at their own discretion.

## CRediT authorship contribution statement

**Sreenivasa Reddy Yeduri:** Conceptualization, Writing – original draft, Writing – review & editing. **Daniel Skomedal Breland:** Software, Methodology, Data curation, Investigation, Visualization. **Om Jee Pandey:** Writing – review & editing, Supervision. **Linga Reddy Cenkeramaddi:** Conceptualization, Supervision, Validation, Writing – review & editing.

## Declaration of Competing Interest

The authors declare that there is no influence from known competing financial interests or personal relationships which have, or could be perceived for the work reported in this article.
